# The 300 versus 300 Study—Low Volume versus High Volume Single Balloon Catheter for Induction of Labor: A Retrospective Case-Control Study

**DOI:** 10.3390/jcm12144839

**Published:** 2023-07-22

**Authors:** Maciej W. Socha, Wojciech Flis, Miłosz Pietrus, Mateusz Wartęga, Monika Szambelan

**Affiliations:** 1 Department of Perinatology, Gynecology and Gynecologic Oncology, Faculty of Health Sciences, Collegium Medicum in Bydgoszcz, Nicolaus Copernicus University, Łukasiewicza 1, 85-821 Bydgoszcz, Poland; 2 Department of Obstetrics and Gynecology, St. Adalbert’s Hospital in Gdańsk, Copernicus Healthcare Entity, Jana Pawła II 50, 80-462 Gdańsk, Poland; 3 Department of Gynecology and Oncology, Jagiellonian University Medical College, 31-501 Kraków, Poland; 4 Department of Pathophysiology, Faculty of Pharmacy, Collegium Medicum in Bydgoszcz, Nicolaus Copernicus University, M. Curie-Skłodowskiej 9, 85-094 Bydgoszcz, Poland; 5 Department of Pharmacology and Therapeutics, Faculty of Medicine, Collegium Medicum in Bydgoszcz, Nicolaus Copernicus University, 85-067 Bydgoszcz, Poland

**Keywords:** cesarean section, delivery, obstetric, labor, induced, pregnancy outcome, urinary catheters, maternal-child nursing, obstetric nursing, midwifery, perinatology, pregnancy, prolonged

## Abstract

The use of a Foley catheter is one of the oldest known methods of labor induction. Therefore, protocols using different volumes of Foley catheter balloons have been developed and tested to accurately determine their effectiveness. In this study, it was decided to retrospectively evaluate two induction of labor (IOL) protocols. The last 300 eligible patients who met the criteria and underwent the low-volume balloon protocol (40–60 mL) IOL were selected. Then next, 300 patients who met the criteria and underwent high-volume balloon (80–100 mL) IOL were selected. Outcomes included time to delivery and parturition type, oxytocin augmentation, operative deliveries and application of intrapartum anesthesia. Overall, the majority of patients delivered within 24 h. Patients who received a high-volume Foley catheter had statistically significantly more vaginal deliveries. The mean-time to delivery in the high-volume catheter group was statistically significantly shorter than in the low-volume catheter group. Patients who received a high-volume Foley catheter required statistically significantly less oxytocin augmentation during induction of labor compared to patients with a low-volume Foley catheter. Regardless of the balloon volume used, the percentage of operative deliveries remained at a similar, low level (8.36% and 2.14%). Regardless of the catheter volume used, the majority of patients chose epidural over intravenous anesthesia. In conclusion, a high-volume balloon Foley catheter IOL is characterized by an increased percentage of vaginal deliveries, shortened time to delivery regardless of the type of delivery, and lower need for oxytocin augmentation.

## 1. Introduction

Induction of labor is currently the most frequently performed operation in modern obstetrics—approximately over 20% of pregnant women undergo induction of labor, with an increasing trend worldwide [1]. Induction of labor is defined as the artificial (iatrogenic) release of uterine contractions leading to vaginal delivery within 24–48 h [2]. Induction of labor is carried out for both fetal and maternal indications. With appropriate decisions and timely intervention, induction of labor can decrease the risk of maternal and fetal mortality and morbidity [3]. In current obstetrical practice worldwide, the standard induction of labor protocol consists of intravenous oxytocin infusion. Additionally, the vast majority of women require prior cervical preparation with a cervical ripening agent due to an unripened cervix. Most commonly, the induction of labor protocol involves a cervical ripening agent followed by intravenous oxytocin infusion. Cervical ripening agents can be broadly divided into two groups: pharmacological and mechanical. Progesterone antagonists and prostaglandins are well-known pharmacological agents, while the Foley and Cook catheters belong to the class of mechanical devices used to ripen the cervix. Each of the agents used for cervical ripening has different efficacy and safety profiles [4,5,6,7]. 

One of the most commonly and one of the oldest used agents for cervical ripening is the Foley catheter. It is characterized by high efficiency and a low percentage of side effects. In addition, it is relatively cheap, which makes it a willingly chosen tool in everyday obstetrical practice [8,9,10]. Despite the long history of the use of the Foley catheter in labor induction, the topic of its effectiveness is still a matter of debate. Despite a fairly simple mechanism of action, its effectiveness may depend on many variables, such as appropriate positioning in the cervical canal or proper fluid filling. It is postulated that the volume to the which the catheter balloon is inflated may significantly affect not only course of induction of labor but also may greatly enhance cervical ripening. The Foley catheter is routinely used for induction of labor at our department. Our clinical observations led to the conclusion that a larger Foley catheter balloon volume may indeed have an effect on labor induction. Therefore, we decided to conduct a retrospective study evaluating the effectiveness of the different volumes of Foley catheter balloons in labor induction.

The aim of this study is to assess the effectiveness of a high-volume Foley catheter compared with a low-volume Foley catheter in achieving vaginal delivery within 24–48 h.

## 2. Materials and Methods

**Figure 1:**
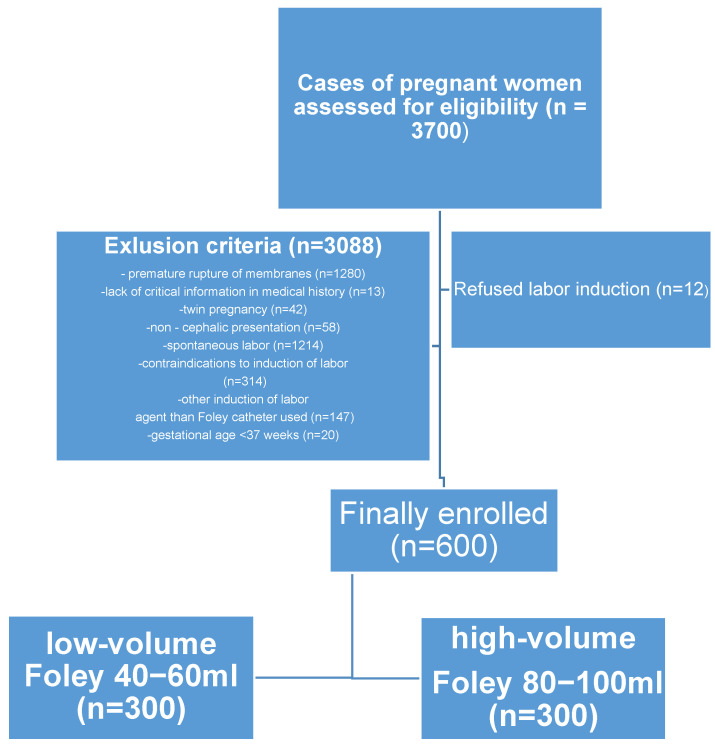
Exclusion and inclusion criteria for the study and division of patients into two groups depending on the volume of the Foley catheter balloon used.

The analysis included medical records of pregnant patients who had a delivery at the Department of Obstetrics and Gynecology, St. Adalbert’s Hospital in Gdańsk (tertiary referral hospital). Based on clinical experience in our department and due to the economic reasons, the protocol of labor induction with a Foley catheter was changed. The use of a Foley catheter with a low-volume balloon which was initially used (40–60 mL) was replaced with a high-volume balloon catheter (80–100 mL). Therefore, it was decided to retrospectively evaluate those two labor induction protocols. For this purpose, a retrospective study was designed where the last 300 eligible patients who met the inclusion criteria and underwent the low-volume balloon induction of labor protocol (40–60 mL) were retrospectively selected. Subsequently, further 300 patients who met the inclusion criteria and underwent high-volume balloon induction (80–100 mL) of labor were additionally selected. Then patients were compared in terms of efficacy of volume of Foley catheter balloon used (Figure 1). Due to the constant number of patients in the groups, the study was called: “The 300 vs. 300 Study”.

The data were generated on the basis of electronic medical records collected by medical personnel during the patient’s stay. Data on the patient and the course of labor were recorded in a computer database by midwives and doctors during and immediately after labor. The obtained dataset was checked for possible errors, and any detected inconsistencies were verified. After analysis of the medical documentation, the following information was obtained: duration of pregnancy (determined on the basis of the date of the last menstruation, confirmed by the first trimester USG), previous obstetric history, type of cervical ripening agent used, parity, course and complications of the current pregnancy, course of the induction of labor, patient’s body-mass index (BMI), duration of labor, route of delivery, and patient demographic data. Informed consent has been obtained from all participants before the start of the induction procedure. All patients were adults. 

Indications for induction of labor were in accordance with the current recommendations of the Polish Society of Gynecologists and Obstetricians [11]. These guidelines list the Foley catheter as one of the main agents used to induce labor and strongly recommend its use due to its low complication rate. However, these recommendations do not specify precisely what balloon volume of the Foley catheter should be routinely used.

The study included only patients qualified for induction of labor for both maternal and fetal indications and with the unprepared cervix (Bishop score > 2 and <6). All patients were in full-term pregnancy (>37 weeks of gestation). Selected patients showed no signs of active labor or any uterine contractions until induction of labor began.

The criteria for inclusion in the study group were: unprepared cervix (Bishop < 6 and >2), single live pregnancy, cephalic fetal presentation, full-term pregnancy (>37 weeks of gestation), unencumbered obstetric history, use of low-volume and high-volume Foley catheter as a cervical ripening agent, and lack of contraindications to vaginal delivery. The exclusion criteria were: onset of spontaneous labor, premature rupture of membranes (PROM), breech fetal position, latex allergy, and any contraindications to vaginal delivery and induction of labor in accordance with the Polish guidelines [11]. 

Induction of labor consisted of the insertion of a Foley catheter into the cervical canal. In reference to our clinical experience, in the study design we adopted two Foley catheter balloon volume ranges (40–60 mL and 80–100 mL) in the labor induction protocol. Once the catheter had passed through the internal os, the balloon was filled with sterile saline to a volume of 40–60 mL or 80–100 mL. The Foley catheter was removed from the cervical canal for the following reasons: the time limit for labor preinduction was reached (24 h); the balloon was expelled spontaneously before the specified time had elapsed; spontaneous rupture of membranes occurred; and women entered the active phase of labor, or fetal distress was encountered. 

In the absence of spontaneously initiated labor (within 24 h), the Foley catheter was removed (regardless of the susceptibility of the cervix), and oxytocin infusion was used in the local protocol for induction of labor. During active phase of labor, the patients were given intrapartum analgesia in the form of an intravenous infusion of Remifentanil or epidural anesthesia on demand. Patients were able to choose the method of anesthesia after having read the educational materials on this topic.

The primary outcome was an assessment of the time from Foley catheter placement to delivery (both cesarean and vaginal) and the percentage of vaginal deliveries. The secondary outcomes were the proportion of women undergoing cesarean section, oxytocin administration, percentage of operative deliveries, and the usage of intrapartum analgesia. We assessed the effectiveness of initiating labor after the administration of a Foley catheter. We compared the groups in terms of the percentage of cesarean sections, and the percentage of vaginal birth with an emphasis on the most common indications for cesarean section and percentage of operative vaginal deliveries. Additionally, we compared the groups in terms of the need for intrapartum analgesia (epidural or intravenous). 

### 2.1. Statistical Methodology

The statistical analyses have been performed using the statistical software StatSoft, Inc. (Street Tulsa, OK, USA) (2014) STATISTICA (version 12.0) and Microsoft Excel 365. The quantitive variables were characterized by the arithmetic mean of standard deviation or median or max/min (range) and 95% confidence interval. The qualitative variables were presented with the use of count and percentage. 

In order to check if a quantitive variable derives from a population of normal distribution, the W Shapiro-Wilk test has been used. Whereas to prove the hypotheses on homogeneity of variances Leven (Brown-Forsythe) test has been utilized.

The statistical significance of differences between the two groups was processed with the t-Student test or U Mann-Withney test. Chi-squared tests for independence were used for qualitative variables. In all of the calculations, the statistical significance level of *p* = 0.05 has been used.

### 2.2. Ethical Approval and Informed Consent

The study was conducted in accordance with the Declaration of Helsinki, and approved by the Institutional Ethics Committee of Collegium Medicum in Bydgoszcz, Nicolaus Copernicus University (KB 226/2023 and annexes obtained 25.04.2023).

## 3. Results

A total of 600 pregnant women (38 to 42 weeks of gestation) who had induction of labor were included in the study. Then, six hundred initially selected patients were divided into two groups (Figure 1) according to the volume of saline-filled Foley balloon used to induction of labor. Three hundred patients received a Foley catheter, in which a balloon was filled with sterile saline to a volume of 40–60 mL (low volume Foley), and the remaining three hundred pregnant women received Foley catheter filled with sterile saline to a volume of 80–100 mL (high volume Foley). The age of patients ranged from 18 to 36 years, with a median age of 28 years (Table 1). During analysis of the data, no adverse effects associated with Foley catheter placement (regardless of volume used) was noted.

The dominant indications for labor induction among selected patients were: gestational diabetes mellitus (GDM), gestational hypertension and preeclampsia, cholestasis of pregnancy, post-term pregnancy, and fetal growth restriction (FGR).

The mean gestational age in the low-volume group was 40.0 (1.5) (range from 38.0–42.0), and the high-volume group was 40.0 (1.5) (range from 38.0–42.0). No statistically significant differences were found (*p* = 0.9944). In the group of patients who received a low-volume balloon catheter, the percentage of primiparas was 49% and 51% of multiparous women. In the group of patients who received the larger-volume balloon catheter, the percentage distribution was 53% primiparous and 47% multiparous, respectively. The mean BMI in the low-volume group was 26 (1.5) (range from 17.0–36.0), and the high-volume group was 27 (1.5) (range from 17.0–36.0). The mean maternal age in the low-volume group was 28 (1.5) (range 18.0–36.0). Respectively in the high-volume group maternal age was 29 (1.5) (range 18.0–36.0). No statistically significant differences were found for those variables and for the induction of labor indications. 

There were two hundred and forty-six vaginal deliveries (82.0%) in the low-volume group, compared to two hundred and sixty-eight (89.3%) in the high-volume group (Table 2). The percentage of vaginal deliveries was statistically significantly higher in the high-volume group compared to the low-volume group (*p* = 0.0104).

There were fifty-four cesarean sections in the low-volume group (18%), while in the high-volume group, there were only thirty-two cesarean sections (10.7%). The cesarean section rate was statistically significantly lower in the high-volume group than in the low-volume group (*p* = 0.0104).

From the total of three hundred patients in the low-volume group, 31.7% (n = 95) delivered within 24 h, and the remaining 68.3% (n = 205) delivered within 48 h as compared to 70.3% (n = 211) and 29.7 (89) in the high-volume group. The time to delivery was statistically significantly shorter in the high-volume group compared to the low-volume group (*p* < 0.0001).

In the low-volume group, the distribution of indications for cesarean section was equal—twenty-seven (50%) patients had cesarean delivery due to nonreassuring fetal heart rate patterns, and the remaining twenty-seven (50%) patients had a lack of labor progression as cesarean section indication (Table 3). Respectively, in the high-volume group, eighteen patients (56.2%) had cesarean section due to nonreassuring fetal heart rate patterns compared to fourteen patients (43.8%) who had a lack of labor progression as a cesarean section indication. No statistical significance was found for these variables (*p* = 0.5749).

The mean time to delivery in the low-volume group was 31.9 h (ranged from 0.5–48.0 h), while in the high-volume group was 20.6 h (ranged from 0.5–48.0 h) (Table 4). The mean time to delivery (both cesarean section and vaginal delivery) was statistically significantly shorter in the high-volume group (*p* < 0.0001).

From the total of three hundred in the low-volume group, 68.3% (n = 205) required oxytocin augmentation, respectively, in the high-volume group, 29.7% (n = 89) (Table 5). Patients in the low-volume group required statistically significantly more oxytocin stimulation in comparison with the high-volume group (*p* < 0.0001).

From the total of three hundred in the low-volume group, 44.7% (n = 134) required epidural anesthesia, and 29.0% (n = 87) required intravenous anesthesia, respectively in the high-volume group, 46.3% (n = 139) and 26.7% (n = 80) (Table 6). No statistically significant differences were found (*p* = 0.5237 and *p* = 0.6819). Overall, of the six hundred participants of the study, four hundred forty (73.3%) required anesthesia, regardless of its type.

Of the total of 246 vaginal deliveries in the low-volume group, 8.51% (n = 21) of patients delivered with a vacuum extractor and 1.84% (n = 5) delivered with forceps (Table 7). Respectively, in the high-volume group, out of 268 vaginal deliveries, 22 (8.18%) patients delivered with vacuum extraction and 6 (2.13%) with forceps. No statistically significant differences were found for those variables (*p* = 0.5326 and *p* = 0.5549).

## 4. Discussion

As mentioned before, one in five women (approximately 20%) undergo labor induction for both maternal and fetal indications. Oxytocin, being the gold standard in labor induction, despite its great efficacy in triggering uterine contractions, has an insignificant effect on cervical ripening. Therefore, the key to a correct and successful induction of labor is proper cervical preparation. The likelihood of successful vaginal delivery after induction of labor is increased if the cervix is favorable (Bishop > 6) [12,13]. Therefore, when patients present for induction of labor and are found to have an unfavorable cervix, cervical ripening is indicated.

As one of the oldest methods to induce labor, mechanical devices were developed to enhance cervical ripening and the onset of labor by dilating the cervix. Currently, the Foley catheter balloon is a commonly used mechanical device for labor induction, which acts not only as a cervical ripening agent but also as a stimulator of endogenous prostaglandins released from the fetal membranes [14]. The use of the Foley catheter in labor induction is highly effective (understood as the percentage of vaginal deliveries) and is a relatively cheap method of labor induction [15,16,17].

Foley catheters with different size balloons have been used with varying success to improve labor induction—40–100 mL [18]. It is postulated that the size of the balloon may significantly affect the maturation of the cervix and thus improve the effectiveness of the induction of labor. The purpose of this study was to evaluate the effectiveness of different balloon volumes (40–60 mL vs. 80–100 mL) to assess the proportion of women who delivered vaginally within 24–48 h.

Our study demonstrated the effectiveness of the use of the Foley catheter in the induction of labor due to the fact that, regardless of the balloon volume used, the vast majority of women delivered within 24 h (51%), most of whom had a vaginal delivery (85.7%). These data are consistent with previously published studies [19,20,21,22,23].

What is extremely interesting is that our study showed that the use of a larger-volume (80–100 mL) balloon catheter (high-volume) was characterized by a statistically significantly higher rate of vaginal deliveries and a lower rate of cesarean sections compared to the low-volume group (89.3% and 10.7% vs. 82% and 18%). Moreover, more than 70% of patients in the high-volume group delivered within 24 h (mean 20.6 h), while in the low-volume group, only 31.7% of patients delivered within this time frame. The majority of patients (68.3%) in the low-volume group delivered within 48 h (mean 31.9 h). Undoubtedly, the use of a larger-volume balloon catheter is more effective in achieving delivery in a shorter time interval. These data are consistent with previous research [24,25,26]. 

The higher percentage of vaginal deliveries and shorter time to delivery in the high-volume group may be due to the duality of the mechanism of action of the Foley catheter. Cervical ripening is a process that consists not only of biochemical and molecular pathways but also of mechanical factors involved. Apart from the mechanical distension of the cervical canal, the Foley catheter, after its proper positioning, causes stretching of fetal membranes and cervical cells, leading to increased secretion of prostaglandin E2 (PGE2), interleukin-8 (IL-8), which are key mediators of cervical ripening. PGE2 also has the ability to directly stimulate uterine contractions. Additionally, cyclic mechanical stretching can greatly increase collagenase activity and hyaluronic acid expression in fibroblasts (which concentration greatly increases in cervical tissue at term) which enhances the influx of water to the cervical stroma leading to collagen fibers reorganization [27,28,29,30]. Therefore, it seems logical to assume that the use of a Foley catheter with a larger-volume balloon results in greater dilation of the cervical canal and also, through a greater mechanical stretch of cervical cells, a greater increase in the concentration of cervical ripening regulators such as PGE2 and IL-8 which leads to greater cervical ripening. 

When considering cesarean sections, in the low-volume group, the lack of labor progression and fetal asphyxia occurred with the same frequency—50%. In contrast, in the high-volume group, in addition to an overall lower cesarean section rate, a lower rate of lack of labor progression, as cesarean section indication, can be observed in comparison with fetal asphyxia (defined as abnormal cardiotocographic record)—43.8% vs. 56.2%. We believe that the lower rate of cesarean sections due to lack of labor progression in the high-volume group is due to better preparation of the cervix with a larger-volume balloon catheter. Referring to previous studies, the use of a catheter with a larger balloon allows for achieving better cervical maturity, which significantly reduces the likelihood of cervical dystocia [14,31,32,33].

Our study also showed that the use of a catheter with a larger balloon volume was associated with a statistically lower need for oxytocin augmentation compared to the low-volume group (29.7% vs. 68.3%, respectively). The aim of our study was to compare two relatively identical groups of patients in whom the same IOL protocol was used with a difference in the volume of the Foley catheter balloon used. The study design assumed that if the uterine contractions did not occur within 24 h of balloon insertion, the Foley catheter was removed and subsequently an oxytocin infusion was administered in accordance with the labor induction protocol adopted in our department. This procedure was conducted independently of the susceptibility of the cervix. Based on our clinical observations using prostaglandin analogs, we have noticed that cervical maturity does not always translate into a Bishop score. In the adopted scheme of induction of labor with a Foley catheter, logistical considerations and the assumption that there were cellular and structural changes in the cervix (pre-induction of labor worked at the cellular level) meant that each patient who did not develop her own contractile function received an oxytocin infusion independently from the susceptibility of the cervix. This distribution of results may again be due to the mechanism of action of the Foley catheter. We believe that the use of a catheter with a larger-volume balloon may have a greater impact on cervical ripening. In addition, it may significantly increase the concentration of endogenous prostaglandins, which translates into triggering contractile activity more effectively [34].

Analyzing the administration of anesthesia during induction of labor, over 70% of the surveyed patients expressed their willingness to undergo this procedure. Much more often, patients (regardless of the balloon volume used) used epidural than intravenous anesthesia (45.5% vs. 27.8%). This is in line with previous studies, which showed the high efficiency of using this type of anesthesia compared to intravenous anesthesia [35,36,37]. In addition, the use of epidural anesthesia was associated with high patient satisfaction [38,39]. Considering individual groups, in each of them, the patients chose epidural anesthesia much more often than intravenous anesthesia. In addition, in both groups, the use of anesthesia remained at similar levels (44.7% and 46.3%). It is also worth noting that in the high-volume group (which was characterized by much faster delivery), the percentage of anesthesia application was at a similar level as in the low-volume group, in which patients had longer period to labor. Based on the above results, it can be concluded that despite the shorter delivery, in the case of the high-volume group, it was not associated with greater pain during delivery. Therefore, we believe that the use of a larger-volume balloon catheter, despite the shorter duration of labor, did not result in a more violent and painful induction of labor.

Considering operative vaginal deliveries, in each study group the percentage of using forceps and the vacuum extractor occurred with similar frequency (i.e., in a small percentage of patients). Of the 514 vaginal deliveries, 8.36% were vacuum extractor assisted and 2.14% obstetrical forceps. However, these data were not statistically significant. These data are consistent with the general statistics of the percentage of operative deliveries [40,41]. These data clearly show that regardless of the balloon volume used, the percentage of operative deliveries did not fluctuate. Moreover, the use of the Foley catheter per se in induction of labor did not lead to an increase in the rate of operative deliveries. With the above in mind, we believe that the Foley catheter can be safely used in labor induction.

Undoubtedly, our study has its limitations. Our study aimed to retrospectively assess the effectiveness of the Foley catheter in labor induction depending on the volume of the balloon used. Patients in both groups were very similar in terms of indications for labor induction, gestational age and parity. In each of the examined groups of patients there were both multiparous and primiparas with a more or less similar percentage distribution. In addition, we did not analyze variables based on the inclusion of patients with a history of caesarean section. A definite limitation of our study is the lack of a thorough analysis of the results depending on the parity of the patients. The assessment of instrumental deliveries depending on the volume of the Foley catheter balloon used was also not analyzed. In addition, no postnatal evaluation of the newborn’s condition was performed, taking into account possible complications. Finally, we believe that an important limitation of our study may be the fact that the analysis included only full-term patients (>37 weeks of gestation) without taking into account the exact division of patients depending on the week of pregnancy. The aim of our study was primarily to assess the effectiveness of the use of the Foley catheter depending on the volume of the balloon used in labor induction. We focused only on the percentage of vaginal deliveries and caesarean sections, while taking into account the use of intrapartum analgesia to accurately assess the effectiveness of the Foley catheter and indirectly asses patient satisfaction understood as the percentage of intrapartum analgesia used. Therefore, we believe that due to some limitations of our study, the results should be interpreted with caution. However, we believe that the results we present may prove to be an excellent starting point for further research in this field (which ought to take into account numerous additional variables). Our study is a retrospective and purely clinical study. We assessed the patients from the clinical point of view and, on this basis, we have drawn our conclusions. We believe that the results we present add a lot to the ongoing discussion regarding the choice of the best methods for labor induction.

## 5. Conclusions

Our retrospective study adds to our understanding of Foley catheter as agents to induce labor. Our study provides evidence for the use of a high-volume Foley catheter (80–100 mL), showing that most patients achieve successful vaginal delivery within 24 h. Moreover, the use of this type of catheter may be associated with a lower caesarean section rate. At the same time, we point out that the shorter time to delivery does not translate into a more violent course, which undoubtedly translates into less extensive use of intrapartum anesthesia. Additionally, the use of a Foley catheter with a larger-volume balloon is not associated with an increased rate of operative deliveries. Finally, we indicate the potential positive effects of using high-volume Foley catheter, which may translate into greater satisfaction of patients after delivery.

In conclusion, we believe that the use of a high-volume Foley catheter has potential positive benefits that may translate into greater patient satisfaction.

## Figures and Tables

**Table 1:** Comparative analysis in two groups: low-volume (40–60 mL) Foley and high-volume (80–100 mL) Foley: study groups characteristics.

	Low-Volume (n = 300)	High-Volume (n = 300)	All (n = 600)	*p*-Value
**Gestational age**				0.9944 ^1^
mean (SD)	40.0 (1.5)	40.0 (1.5)	40.0 (1.5)	
range	38.0–42.0	38.0–42.0	38.0–42.0	
median (IRQ)	40.0 (2.0)	40.0 (2.0)	40.0 (2.0)	
95%CI	[39.8; 40.2]	[39.8; 40.2]	[39.9; 40.1]	
**Maternal Age**				0.8324 ^1^
Mean (SD)	28 (1.5)	29 (1.5)	28 (1.5)	
Range	18.0–36.0	18.0–36.0	18.0–36.0	
**BMI**				0.5553 ^1^
Mean (SD)	26 (1.5)	27 (1.5)	26 (1.5)	
Range	17.0–36.0	17.0–36.0	17.0–36.0	
**Parity**				0.1652 ^1^
Primiparas	147 (49.0%)	159 (53.0%)	306 (51.0%)	
Multiparas	153 (51.0%)	141 (47.0%)	294 (49.0%)	
Parity range	1–7	1–7	1–7	
**IOL Indications**				0.8894 ^1^
GDM	56 (18.6%)	58 (19.4%)	114 (19.0%)	0.8354 ^1^
Hypertension	48 (16.0%)	44 (14.6%)	92 (15.3%)	0.6510 ^1^
Preeclampsia	41 (13.6%)	40 (13.4%	81 (13.6%)	0.9051 ^1^
Cholestasis	51 (17.1%)	49 (16.3%)	100 (16.6%)	0.8269 ^1^
Post-Term pregnancy	65 (21.6%)	68 (22.7%)	133 (22.2%)	0.8204 ^1^
Fetal Growth Restriction	39 (13.1%)	41 (13.6%)	80 (13.3%)	0.8105 ^1^

^1^ U Mann-Whitney.

**Table 2:** Comparative analysis in two groups: low-volume (40–60 mL) Foley and high-volume (80–100 mL) Foley: time to delivery and parturition type.

	Low-Volume (n = 300)	High-Volume (n = 300)	All (n = 600)	*p*-Value
**Time to delivery**				<0.0001 ^1,2^
within 24 h	95 (31.7%)	211 (70.3%)	306 (51.0%)	
within 48 h	205 (68.3%)	89 (29.7%)	294 (49.0%)	
**Parturition Type**				0.0104 ^2^
Vaginal delivery	246 (82.0%)	268 (89.3%)	514 (85.7%)	
Cesarean section	54 (18.0%)	32 (10.7%)	86 (14.3%)	

^1^ U Mann-Whitney; ^2^ Chi-square.

**Table 3:** Comparative analysis in two groups: low-volume (40–60 mL) Foley and high-volume (80–100 mL) Foley: cesarean section and indications for cesarean delivery: lack of labor progression and nonreassuring fetal heart rate patterns.

	Low-Volume (n = 54)	High-Volume (n = 32)	All (n = 86)	*p*-Value
**Cesarean section indication**				0.5749 ^1^
Nonreassuring fetal heart rate patterns	27 (50.0%)	18 (56.2%)	45 (52.3%)	
Lack of labor progression	27 (50.0%)	14 (43.8%)	41 (47.7%)	

^1^ Chi-square.

**Table 4:** Comparative analysis in two groups: low-volume (40–60 mL) Foley and high-volume (80–100 mL) Foley: time to delivery.

	Low-Volume (n = 300)	High-Volume (n = 300)	All (n = 600)	*p*-Value
**Time to delivery**				<0.0001 ^1^
mean (SD)	31.9 (13.8)	20.6 (11.2)	26.3 (13.7)	
range	0.5–48.0	0.5–48.0	0.5–48.0	
median (IRQ)	37.0 (23.0)	20.0 (17.0)	24.0 (24.0)	
95%CI	[30.3; 33.4]	[19.4; 21.9]	[25.2; 27.4]	

^1^ U Mann-Whitney.

**Table 5:** Comparative analysis in two groups: low-volume (40–60 mL) Foley and high-volume (80–100 mL) Foley: oxytocin augmentation.

	Low-Volume (n = 300)	High-Volume (n = 300)	All (n = 600)	*p*-Value
**Oxytocin**				<0.0001 ^1^
	205 (68.3%)	89 (29.7%)	294 (49.0%)	

^1^ Chi-square.

**Table 6:** Comparative analysis in two groups: low-volume (40–60 mL) Foley and high-volume (80–100 mL) Foley: application of epidural anesthesia and intravenous anesthesia.

	Low-Volume (n = 300)	High-Volume (n = 300)	All (n = 600)	*p*-Value
**Epidural anesthesia**				0.6819 ^1^
	134 (44.7%)	139 (46.3%)	273 (45.5%)	
**Intravenous anesthesia**				0.5237 ^1^
	87 (29.0%)	80 (26.7%)	167 (27.8%)	

^1^ Chi-square.

**Table 7:** Comparative analysis in two groups: low-volume (40–60 mL) Foley and high-volume (80–100 mL) Foley: operative vaginal deliveries (vacuum extractor and forceps).

	Low-Volume (n = 246)	High-Volume (n = 268)	All (n = 514)	*p*-Value
**Vacuum extractor (VE)**				0.5326 ^1^
	21 (8.51%)	22 (8.18%)	43 (8.36%)	
**Obstetrical forceps (F)**				0.5549 ^1^
	5 (1.84%)	6 (2.13%)	11 (2.14%)	

^1^ Chi-square.

## Data Availability

The data presented in this study are available on request from the corresponding author. The data are not publicly available due to privacy restrictions.
